# Development of an amphibian sperm biobanking protocol for genetic management and population sustainability

**DOI:** 10.1093/conphys/coac032

**Published:** 2022-05-23

**Authors:** Isabella J Burger, Shaina S Lampert, Carrie K Kouba, Dana J Morin, Andrew J Kouba

**Affiliations:** Department of Wildlife, Fisheries and Aquaculture, Mississippi State University, Mississippi State, MS, 39762, USA; Department of Biochemistry, Molecular Biology, Entomology, and Plant Pathology, Mississippi State, MS, 39762, USA; Department of Biochemistry, Molecular Biology, Entomology, and Plant Pathology, Mississippi State, MS, 39762, USA; Department of Biochemistry, Molecular Biology, Entomology, and Plant Pathology, Mississippi State, MS, 39762, USA; Department of Wildlife, Fisheries and Aquaculture, Mississippi State University, Mississippi State, MS, 39762, USA; Department of Wildlife, Fisheries and Aquaculture, Mississippi State University, Mississippi State, MS, 39762, USA

**Keywords:** model species, genome banking, freezing rate, cryoprotectant, assisted reproductive technologies

## Abstract

Sperm cryopreservation is a vital tool in amphibian assisted reproductive technologies that aids in genetic and population management, specifically for at-risk species. Significant advancements have been made in the cryopreservation of amphibian sperm, yet there is little information on how the cryopreservation process influences fertilization and embryonic development. In this study, we tested several cryoprotective agents (CPAs) and freezing rates on sperm recovery, fertilization potential and embryo development using Fowler’s toads (*Anaxyrus fowleri*) as a model amphibian species for application to at-risk anurans. Three cryoprotectant treatments were tested, which included 10% trehalose + 0.25% bovine serum albumin with (1) 5% N,N-dimethylformamide (DMFA); (2) 10% DMFA; or (3) 10% dimethyl sulfoxide (DMSO). Additionally, sperm in each cryoprotectant was frozen at two different rates, −32 to −45°C/min and −20 to −29°C/min. Post-thaw sperm analysis included motility, morphology, viability, fertilization success and embryo development. Results show that 10% DMFA produced significantly higher (*P =* 0.005) post-thaw sperm motility than 5% DMFA and was similar to 10% DMSO. Furthermore, sperm frozen at −32 to −45°C/min had significantly higher post-thaw motility (*P* < 0.001) compared to sperm frozen at −20 to −29°C/min. We also found that embryos fertilized with sperm frozen with 5% DMFA resulted in significantly higher (*P* = 0.02) cleavage than 10% DMSO, yet there was no other effect of CPA on fertilization or embryo development. Furthermore, embryos fertilized with sperm frozen at −32 to −45°C/min resulted in significantly higher cleavage (*P* = 0.001), neurulation (*P* = 0.001) and hatching (*P* = 0.002) numbers than sperm frozen at a rate of −20 to −29°C/min. Overall, eggs fertilized with frozen–thawed sperm produced 1327 tadpoles. These results provide insight towards a biobanking strategy that can be applied to imperilled species to preserve genetic lineages and bolster offspring genetic diversity for reintroduction.

## Teaser

Sperm cryopreservation is a crucial tool in amphibian assisted reproductive technologies that aids in population and genetic management of at-risk species. Using *Anaxyrus fowleri* as a model species, this study found that fertilization success and embryonic development are dependent on the cryoprotectant and freezing rate applied to sperm during cryopreservation.

## Introduction

Sperm cryopreservation requires very low temperatures to preserve spermatozoa for the short or long term, typically for application towards future reproductive efforts and genetic management (Medeiros *et al.,* 2002; Pegg, 2015). This technique, along with the transport of frozen semen, has been used heavily for production management in livestock, poultry and aquaculture (Gee *et al.,* 2004; Ugur *et al.,* 2019; Viveiros *et al.,* 2012), for genetic management in the equine industry (Moore *et al.,* 2005) and to address human infertility (Mocé *et al.,* 2016). Although there has been a high level of success in offspring production and genetic management in the above cases, there are still aspects of the cryopreservation process that can have deleterious effects on spermatozoa, affecting the quality of the sperm, fertilization potential of eggs and even the survivability of produced offspring.

The freezing and thawing processes applied during cryopreservation can cause morphological and functional damage to the sperm cell, affecting post-thaw survivability and subsequent fertilization (Bailey *et al.,* 2003; Vajta and Kuwayama, 2006). Cell structural damage includes membrane degradation, detachment or impairment of the head and/or tail and DNA fragmentation, all of which can be caused by the physical stressors of ice crystal formation and osmotic shock, or chemical stressors of cold shock induced apoptosis and reactive oxygen species generation (Fitzsimmons *et al.,* 2009; Kopeika *et al.,* 2015; Vajta and Kuwayama, 2006). To moderate the deleterious effects of cryopreservation on spermatozoa, cryoprotective agents (CPAs) can be added to offset ice crystal formation by increasing the solute concentration of the extracellular environment, thus increasing the rate of cell dehydration (Kopeika *et al.,* 2015; Mazur, 1970; Pegg, 2015). Furthermore, cooling rates can be altered to mitigate ice crystal formation as well as minimize osmotic and cold shock through controlled extracellular and intracellular fluid exchange (Vajta and Kuwayama, 2006; Kopeika *et al.,* 2015). Numerous protocols and combinations of cryoprotectants and freezing rates have been applied in sperm cryopreservation for domestic mammals, poultry and common amphibian species to determine a protocol of best fit for these groups. Now, building on this foundation, sperm cryopreservation has expanded beyond commercial use and is increasingly being applied to endangered species conservation.

As of 2021, there are over 40 000 species that are threatened with extinction, with amphibians being the most endangered taxa. Currently, ~ 3% (184) of all amphibian species are extinct, and another 41% (2442) are at risk (IUCN, 2021). To mitigate this extinction threat, zoos and aquariums are establishing captive breeding programmes that focus on sustainability and increasing reproductive output for reintroductions, while trying to maintain genetic diversity of these species (Griffiths and Pavajeau, 2008; Harding *et al.,* 2016; Howell *et al.,* 2020; Zippel *et al.,* 2011). There are various environmental stimuli that amphibians require to reproduce, and the lack of these cues in captive settings has led to poor natural breeding levels (Kouba *et al.,* 2009). To address the low reproductive output, various alternative management strategies are being implemented, including hormone therapy, sperm cryopreservation, and *in vitro* fertilization (IVF), in order to bolster reproductive success and maintain or increase genetic variation of both *in situ* and *ex situ* populations (Burger *et al.,* 2021; Clulow *et al.,* 2014; Kouba and Vance, 2009).

Amphibian sperm can be collected through exogenous hormone therapy using live animals (Burger *et al.,* 2021; Della Togna *et al.,* 2017; Uteshev *et al.,* 2013), from testes macerates excised from euthanized animals (Mansour *et al.,* 2010; Upton *et al.,* 2018), or from cold-stored carcasses (Kaurova *et al.,* 2021). Even though there has been success using different sources of sperm for cryopreservation, the resulting post-thaw sperm data are highly variable. Multiple studies have looked at how different cryopreservation protocols affect post-thaw sperm motility and report varying levels of success, ranging from 1 to 65% motility (Browne *et al.,* 2002; Poo and Hinkson, 2019; Shishova *et al.,* 2011). In these three studies alone, eleven different cryoprotectant treatments were applied, with no overlapping treatments between experiments. This high level of variation across studies justifies the need to standardize existing sperm cryopreservation protocols for application to a broad suite of amphibian species. In order to establish a more universal cryopreservation protocol, a common animal model of similar phylogeny to targeted at-risk species can be used to test various methodologies to determine how these procedures affect post-thaw sperm parameters, fertilization success and embryo development.

Model species are defined as organisms that are studied to increase our knowledge of certain biological processes that can then be applied to other species (Leonelli and Ankeny, 2013). Appropriate models properly represent the target group(s), are easy to breed and house in large numbers and have rapid maturity rates, high fecundity and short life spans (Barr, 2003; Leonelli and Ankeny, 2013). Currently, the most common amphibian models *Xenopus spp.* and *Axoltl* are fully aquatic species that are primarily used for medical research (Buchholz *et al.,* 2006; Tilley *et al.,* 2021). Because they are fully aquatic, these two animals are not representative of a majority of all amphibian species, as many are terrestrial or semi-aquatic. We propose the common Fowler’s toad (*Anaxyrus fowleri)* can serve as a model for imperilled terrestrial anurans due to the morphological and phylogenic similarities it shares with many US anurans of conservation concern, including the Puerto Rican crested toad (*Peltophryne lemur*), Houston toad (*Anaxyrus houstonensis*), Wyoming toad (*Anaxyrus baxteri*), Yosemite toad (*Anaxyrus canorus*) and Arroyo toad (*Anaxyrus califorinucus*).

The purpose of this study was to use the Fowler’s toad as a model species to test the effect of various sperm cryopreservation protocols on sperm quality, fertilization success, and embryonic development. Specific objectives include (i) determining the effect of three cryoprotectants on post-thaw sperm motility, fertilization capacity and embryo development; (ii) determining the effect of two freezing rates on post-thaw sperm motility, fertilization capacity and embryo development; and (iii) evaluating the optimal cryopreservation protocol on sperm post-thaw viability and morphology compared to pre-freeze values. Cryopreservation protocols developed from this study have been applied to other threatened amphibian species to test the transferability of this technology for conservation as well as establish the Fowler’s toad as a suitable model species for studying cryobiology in anurans (Burger, unpublished data).

## Methods

### Animals

Fowler’s toads were collected during the summer of 2020 (Mississippi Department of Wildlife, Fisheries and Parks permit No. 0504202) from Oktibbeha County, Mississippi (33.3887°N, 88.9031°W). All toads were housed in same-sex groups of 2–6 individuals in ventilated polycarbonate containers (30 L × 46 W × 66 H cm). Enclosures were fitted with shelter and water, and temperatures maintained at 20–23°C. Animals were fed a varying diet of crickets, mealworms, and Dubia roaches three times a week. Insects were gut-loaded with Repashy SuperLoad (Repashy Ventures Inc., CA, USA) and dusted with a vitamin D supplement prior to feeding. All animal procedures were approved by the Mississippi State University Institutional Animal Care and Use Committee (IACUC-19-345).

### Hormone administration, sperm collection and sperm analysis

To collect spermic urine, male toads (*n* = 32) were administered 300 IU of human chorionic gonadotropin (hCG; Sigma-Aldrich, Product no. CG5) via intraperitoneal (IP) injections as previously described (Kouba *et al.,* 2012). Toads were then isolated in 2-litre plastic tubs with ~1.5 cm of water in the bottom to stimulate water uptake and urine production. Spermic urine was collected from each male at 2, 3, 5 and 7 h post-hormone administration by holding the individual over a petri dish until urination occurred. The samples were placed in sterile 1.5-ml Eppendorf tubes and immediately analysed for sperm motility, quality and concentration. Urine was collected at multiple time points to increase the volume of sperm that could be cryopreserved in multiple treatment groups. Eppendorf tubes containing spermic urine were stored at 4°C during and post-analysis until the final collection, where samples collected at the various time points were pooled for Experiment 1*.*

The following sperm motility parameters were measured immediately after each collection: forward progressive motility (FPM; sperm exhibiting flagellar movement and progressing forward), non-progressive motility (NPM; sperm exhibiting flagellar movement, but not progressing forward) and non-motile sperm (NM; sperm with no flagellar movement). Total motility was determined as the sum of FPM and NPM (Julien *et al.,* 2019). The quality of FPM (QFPM) was also measured on a 0–5 scale, with 0 representing no forward movement of the sperm and 5 representing forceful rapid forward movement. The above parameters were analysed by placing 12 μl of the sperm sample under a 40× objective on an Olympus CX43 phase-contrast microscope following each collection. Concentration was measured by inactivating the sperm at either a 1:1, 1:5 or 1:10 ratio of spermic urine to phosphate-buffered saline (PBS), depending on density of the sperm. Sperm were counted using a Neubaeur Haemocytometer (Hausser Scientific, product no. 3200). The total number of sperm in a sample was determined by multiplying the overall concentration by the volume (millilitre) of the sample, and total motile sperm was determined by multiplying the total number of sperm by the TM. To determine an overall assessment rating of the sperm, a sperm motility index was calculated for each sample using the following equation: [(QFPM x 20) + (%TM)]/2 (Howard *et al.,* 1990). Osmolality of spermic urine was measured using the Vapro55204 osmometer.

### Experiment 1: Effect of different cryoprotectants and freezing rates on post-thaw sperm motility and fertilization success

After the final 7 h collection, spermic urine from each individual was pooled and analysed. From the individual pooled sperm, 100 μl aliquots were mixed with one of three CPA treatment groups (*n* = 22 toads/treatment). The three treatment groups contained a base of 10% trehalose + 0.25% bovine serum albumin (BSA) with (i) 5% DMFA, (ii) 10% DMFA or (iii) 10% DMSO. To reach the desired CPA treatment concentrations, sperm aliquots were mixed 1:1 with 100 μl of a two times stock.

Additionally, each of the three treatment groups was divided into two subgroups to evaluate freezing rates: (i) straws held at 5 cm above liquid nitrogen (LN_2_) prior to plunging (−32 to −45°C/min) and (ii) straws held at 10 cm above LN_2_ prior to plunging (−20 to −29°C/min). At least two replicates per treatment and sub-treatment group, per individual, were conducted to further reduce variability. In order to test all three treatments and subgroups, > 1.2 ml of spermic urine per individual was needed; hence, the pooling over several collection time points. Once sperm were combined with the CPA treatments, the 200 μl cryosuspensions were loaded into 0.25 ml plastic freezing straws (Minitube International, Germany) and plugged with Critoseal (Leica Biosystems, IL, USA). The preparation and loading of the cryosuspensions into the straws took place on an ice pack in order to maintain the temperature at ~4°C while handling. Straws loaded with cryosuspension were then equilibrated in the refrigerator at 4°C for 10 min, prior to the freezing process.

For straws frozen at −32 to −45°C/min, the holding rack was removed from the refrigerator and placed into a freezing unit (30 × 24 × 15 cm; Minitube International, Germany, product no. 15043/0636) 5 cm above LN_2_. Straws were held for 10 min in the LN_2_ vapor before plunging into the liquid. For straws frozen at −20 to −29°C/min, the straws were removed from the refrigerator and transferred to a Styrofoam box (21 × 16.5 × 20.5 cm) with a rack situated 10 cm above LN_2_. Similar to above, straws were held for 10 min in the LN_2_ vapor before plunging into the liquid. Once equilibrated, straws were removed and placed into a goblet, which was immediately transferred into a long-term cryopreservation storage tank. Straws remained in the genome bank for up to 11 months until the thawing process and post-evaluation.

The thawing process consisted of removing individual straws from the cryopreservation tank and submerging them into a 40°C water bath until all ice crystals melted (~ 5 s). The thawed contents were expelled onto a plastic petri dish and diluted at a 1:10 ratio of frozen–thawed sperm to sterile embryo transfer water. This dilution activated the frozen sperm in the sample, which was then analysed immediately after dilution. To determine the effects of the cryoprotectant treatments and freezing rates on post-thaw sperm motility, we measured all motility variables as described above for pre-freeze analysis. Both absolute motility and relative motility were recorded for all post-thaw sperm samples. Absolute motility is the raw motility values counted during post-thaw sperm analysis, whereas relative motility is the ratio of post-thaw to pre-freeze motility. Relative motility takes into account the starting motility of the sample and adjusts the final value to represent how much sperm motility was recovered after the freezing process.

### *In vitro* fertilization

**Figure 1:**
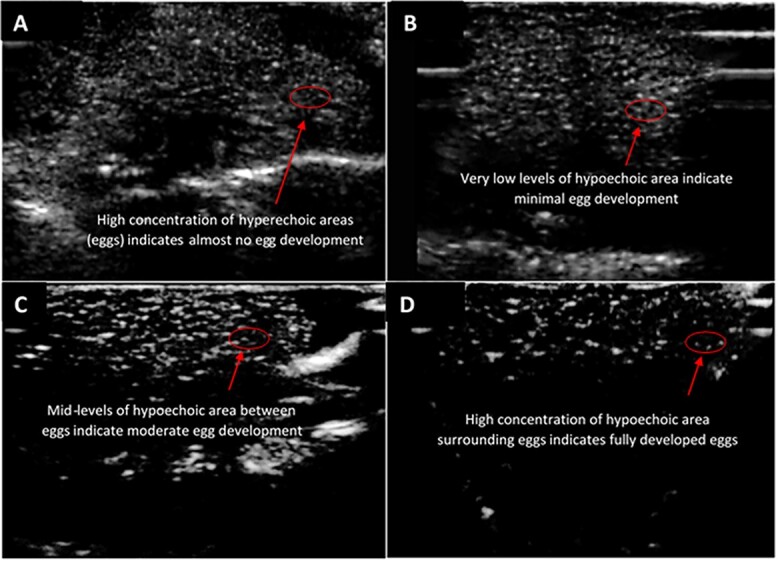
Representative images of grades 0–3 of Fowler’s toads. Egg jelly is indicated by hypoechoic (dark) areas and eggs by hyperechoic (white) areas. (A) Almost no hypoechoic areas between eggs indicate this female has almost no egg development. (B) Low levels of hypoechoic areas between hyperechoic areas indicate minimal egg development. (C) Mid-levels of hypoechoic are between eggs show that this female has moderate egg development and could be administered a priming dose, followed by an ovulatory dose for IVF. (D) High levels of hypoechoic area indicate that this female has fully developed eggs and could be administered an ovulatory dose for IVF. Females at ultrasound grades 2 and 3 were selected for IVF.

Ultrasonography was used to select females for IVF according to follicle maturation and development as previously described for *Lithobates sevosa* (Graham *et al.,* 2018). All ultrasound exams were graded on a 0–3 scale, with 0 being no egg development and 3 being an even level of development across the entire ovary. No egg development is determined by a lack of hypoechoic space interspersed in the hyperechoic areas. As seen in the red circle in Fig. 1A, hyperechoic space represents the egg follicles, and the hypoechoic area represents the egg jelly surrounding the follicles. High levels of hypoechoic space indicate that a female has fully developed eggs and could be administered a hormonal ovulatory dose to stimulate oviposition (Bronson and Vance, 2019). As ultrasound ovarian development images have never been published for Fowler’s toads, we present here a guide to follicle development that can be used to select females for ovulation (Fig. 1). From our captive population, eleven females of grades 2–3 were selected for hormone stimulation and IVF. Females at grade 2 were administered a prime of 100 IU hCG, followed by a second prime of the same hormone concentration 72 h later. Twenty-four hours after the second prime, an ovulation dose of 300 IU hCG + 15-ug gonadotropin-releasing hormone analogue (GnRHa; Sigma-Aldridge, product no. L4513) was administered. Females at grade 3 only received one prime, followed by the ovulatory dose 24 h later. Once the ovulatory dose was administered, the female was placed in ~1 cm of water to encourage egg laying. Females were checked at regular intervals until a few eggs were observed to have been spontaneously laid in the plastic tubs. If 12 h passed without any laying, eggs were expressed using gentle pressure to the abdomen as described by Bronson and Vance (2019). Once the female started laying, about 100 eggs were placed in separate dishes for the fertilization experiments using the cryopreserved sperm treatments.

A sub-group of cryopreserved sperm from male toads (*n* = 6) was selected to use in IVF attempts to determine the effects of the CPA treatments and freezing rates on fertilization and embryo development. For IVF, cryopreserved sperm from the six males were thawed as described above, reactivated in embryo transfer water and analysed for the same parameters as previously detailed. Reactivated sperm were pipetted over the eggs and allowed to sit for 5 min before flooding the dish with aged tap water. Experimental controls consisted of parthenogenetic controls for each male–female pairing to establish the level of auto-activation that may have occurred during egg expression and fresh sperm controls to ensure that egg quality was not a factor on experimental variables. Embryonic development was monitored to determine the number of individuals to reach Gosner stages 3 (cleavage), 13 (neurula) and 20 (hatched) (Gosner, 1960).

Once individuals reached Gosner stage 20, undeveloped eggs were removed and the tadpoles were relocated to larger plastic containers (18 × 28 × 14 cm), keeping individuals separated based on treatment type. Air pumps and air stone cylinders were added to the tanks to oxygenate the water. Tanks were spot cleaned every day, and water was changed every other day. Tadpoles were fed a mixture of Tetra Tetramin Tropical Flakes and Tetra Pleco Wafers until their release.

### Experiment 2: effect of optimal cryoprotectant and freezing rate on sperm viability, structural integrity and morphology

Because the protocol of freezing sperm with 10% DMFA at 5 cm above LN_2_ produced the highest level of post-thaw motility and fertilization rates in Experiment 1*,* we wanted to further determine this treatment’s effect on sperm physiology, specifically impacts to viability, structural integrity and morphology that occurred during cryopreservation or thawing. For Experiment 2, males (*n* = 10) were administered hormone in the same method as described above, and collected sperm were analysed for pre-freeze variables including motility, concentration, viability, abnormality and presence/absence of the mitochondrial vesicle (MV).

Sperm viability was measured using the SYBR-14/propidium iodine (PI) dual viability stain that measures live/dead sperm. SYBR-14 and PI were thawed and separated into 1- and 2 μl aliquots, respectively. SYBR-14 was diluted 1:50 in a Holtfreter’s solution, and 5 μl of this mixture was added to 5 μl of spermic urine. Following a 7 min incubation period at room temperature, 2 μl of PI was added to the 10 μl spermic urine/SYBR-14 mixture. After another seven-minute incubation period, the 12 μl suspension was placed on a slide, cover slipped and evaluated on a Nikon Eclipse N*i* microscope with a red/green filter. One hundred sperm were counted as live or dead, with red cells representing dead sperm and green cells representing live sperm (Figure 4C−E). Staining protocols followed those outlined in Langhorne (2016). For pre-freeze and post-thaw morphology analysis, sperm were categorized into four groups: (i) no abnormalities, (ii) abnormal tail (tails are split or curled), (iii) abnormal head (heads are curled, engorged, broken, bent or absent) and (iv) both head and tail abnormalities (Fig. 6B−E). The presence/absence of the MV was also categorized into three groups: (i) motile sperm + MV, (ii) nonmotile sperm + MV and (iii) nonmotile sperm + MV (Fig. 5C and D). For both the abnormality and MV measurements, 12 μl of spermic urine were placed on a slide and a sampling of 100 random spermatozoa was counted.

As described above, spermic urine from the additional 10 males was frozen using a final concentration of 10% DMFA suspended at 5 cm above LN_2_. Similarly, the spermic urine was prepared for cryopreservation, frozen and thawed as described above. The same motility (FPM, NPM, NM and TM), viability (live, dead), abnormality (normal, tail, head or both) and MV (motile + MV, motile – MV, nonmotile + MV, nonmotile – MV) parameters were measured post-thaw and compared to pre-freeze values in order to determine the effect of cryopreservation on these various factors.

### Statistical analysis

To determine an effective sampling size prior to experimentation, we ran a power analysis with a statistical significance of 0.05, a power of 0.8 and an effect size of 0.5 (Sullivan and Feinn, 2012). After exploratory data analysis, we decided to analyse all motility and embryo development data using Generalized Additive Model for Location Scale and Shape (GAMLSS) tests. Absolute and relative sperm motility was run under a beta distribution, and FPM and all development data (cleavage, neurulation and hatch) were run under a zero-inflated beta distribution. All females responded to the hormone treatment (*n* = 11), yet data from six were included in the analysis. The other five females did not produce enough eggs for all six treatments to be applied, so they were removed from the analyses to control for any potential bias and variation. For motility and fertilization data, we evaluated candidate models using freezing rate, CPA and the interaction between freezing rate and CPA as fixed effects and the individual (or male–female pair for embryo development data) as a random effect. Candidate models for cleavage data also included post-thaw total motility to determine if motility had an effect on initial fertilization success. We measured the relative support of each model, including all single-variable models, additive combinations and interactions, using Akaike information criterion (AIC). Highly competitive models were those with a ΔAIC < 2 from the top model. Comparisons between pre-freeze and post-thaw MV and abnormal tail parameters were analysed using paired Wilcoxon Signed-Rank tests. Comparisons between pre-freeze and post-thaw viability, total motility and the remaining abnormality parameters were analysed using paired sample *t* tests. Effect size for data run with a paired Wilcoxon Signed-Rank tests is represented as Cohen’s *d*. Prior to analyses, data were tested for normality using Shapiro–Wilk tests and residual plots, and homogeneity was tested using Levene’s tests. Statistical significance was set at 0.05, and data are expressed as mean ± SEM. All data was analysed in RStudio with R version 4.0.2.

## Results

### Fresh sperm parameters following exogenous hormone administration

All males (*n* = 32) responded to hormone treatment and produced a sufficient quality and quantity of sperm for cryopreservation. Total volume of the fresh, pooled sperm samples ranged from 1.2 to 2.3 ml (mean = 1.6 ± 0.1 ml), and concentration ranged from 0.8 to 28.6 × 10^6^ sperm/ml (mean = 4.9 × 10^6^ sperm/ml). From these values, we were able to determine the total number of sperm present in each pooled sample, which ranged from 1.0 to 13.9 × 10^6^ sperm (mean = 3.5 ± 0.4 × 10^6^ sperm) across all males. Total motility varied from 53% to 95% (mean = 81 ± 2%), and out of this total, 23% to 84% (mean = 54 ± 3%) of sperm were forward progressive with a QFPM ranging from 2 to 5 (mean = 3.5 ± 0.1). The QFPM and total motility were used to determine the sperm motility index for each individual, which ranged from 20.3 to 50.4 (mean = 35.4 ± 1.5) across all males. Table 2 shows the average spermic urine pre-freeze values for volume, osmolality, FPM, QFPM, TM, sperm motility index, concentration, total sperm and total motile sperm.

### Experiment 1: effect of different cryoprotectants and freezing rates on post-thaw sperm motility and fertilization success

Experiment 1 evaluated how the three cryoprotectant treatments (5% DMFA, 10% DMFA and 10% DMSO) and two freezing rates (−32 to −45°C/min and −20 to −29°C/min) affected post-thaw sperm motility, egg fertilization and embryo development. For post-thaw total sperm motility, there was considerable support for one model (CPA + freezing rate; *w_i_* = 0.75), with no other models that were highly competitive (ΔAIC < 2) (Table 1), suggesting that both cryoprotectant and freezing rate affected post-thaw sperm motility. In comparison, model analysis for embryo development (cleavage, neurulation and hatching) showed that all highly competitive models (ΔAIC < 2) included freezing rate, but not all included cryoprotectant (Table 1), indicating that the varying freezing rates had a greater effect on embryo development than the different cryoprotectants. Furthermore, for cleavage, the most competitive model included total motility as an additive effect, along with freezing rate and CPA (ΔAIC = 0.00, *w_i_ =* 0.61), followed by a model with an additive effect with just CPA and freezing rate (ΔAIC = 1.36, *w_i_* = 0.37). These results showed that the total motility of sperm, cryoprotectant and freezing rate affected the percent cleavage (Table 1). For percent neurulation and hatching, the competitive models were the ones with an additive effect (CPA + freezing rate; ΔAIC_neurula_ = 0.26, *w*_*neurula* =_ 0.41, ΔAIC_hatch_ = 1.60, *w_hatch_* = 0.28) and the model with only freezing rate as an explanatory variable (ΔAIC_neurula_ = 0.00, *w_neurula_* = 0.46, ΔAIC_hatch_ = 0.00, *w_hatch_ =* 0.62) (Table 1). This result indicates that even though both cryoprotectant and freezing rate affected neurulation and hatching, the freezing rate had a much stronger effect than the cryoprotectants.

**Table 1:** Results of AIC analysis for models of Fowler’s toad post-thaw total motility and embryo development rates

**Response Variable**	**Candidate models**	**d*f***	**AIC**	**▲ AIC**	**Weight (*w***_***i***_ **)**
**Total motility**	Freezing rate + cryoprotectant + random (individual)	26	−382.10	0.00	0.75
	Freezing rate + cryoprotectant + freezing rate ^*^ cryoprotectant + random (individual)	28	−378.93	3.18	0.15
	Freezing rate + random (individual)	24	−377.90	4.20	0.09
	Cryoprotectant + random (individual)	24	−362.32	19.78	0.00
	Random (individual)	8	−64.54	317.56	0.00
**Cleavage rate**	Freezing rate + cryoprotectant + total motility + random (round)	13	−113.41	0.00	0.61
	Freezing rate + cryoprotectant + random (round)	13	−112.05	1.36	0.37
	Freezing rate + cryoprotectant + freezing rate ^*^ cryoprotectant + random (individual)	11	−109.06	4.35	0.07
	Freezing rate + random (round)	15	−106.08	7.33	0.02
	Cryoprotectant + random (round)	12	−100.61	12.80	0.00
	Random (round)	4	−25.57	87.84	0.00
**Neurulation rate**	Freezing rate + random (round)	12	−115.11	0.00	0.46
	Freezing rate + cryoprotectant + random (round)	14	−114.85	0.26	0.41
	Freezing rate + cryoprotectant + freezing rate ^*^ cryoprotectant + random (round)	16	−112.55	2.57	0.13
	Cryoprotectant + random (round)	13	−105.19	9.92	0.00
	Random (round)	8	−18.12	66.99	0.00
**Hatch rate**	Freezing rate + random(round)	11	−107.73	0.00	0.62
	Freezing rate + cryoprotectant + random (round)	13	−106.13	1.60	0.28
	Freezing rate + cryoprotectant + freezing rate ^*^ cryoprotectant + random (round)	15	−107.73	3.81	0.09
	Cryoprotectant + random (round)	12	−97.96	9.77	0.00
	Random (round)	9	−66.40	41.33	0.00

**Figure 2:**
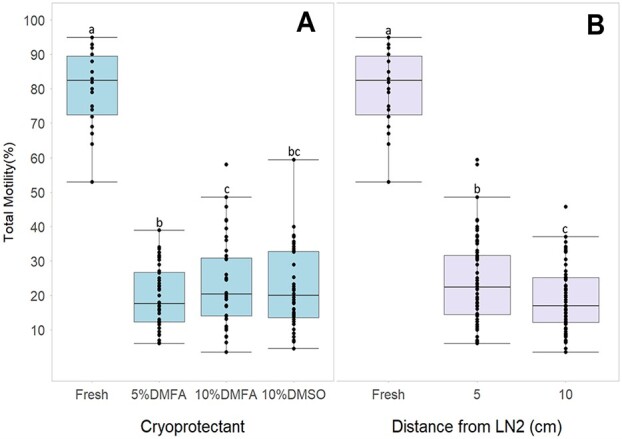
Post-thaw sperm total motility separated by cryoprotectant and freezing rate. (A) Percentage of post-thaw total sperm motility separated by cryoprotectant added to the fresh sperm upon freezing. (B) Percentage of total post-thaw sperm motility depending on freezing rate, shown as distance from LN_2_. Five cm from LN_2_ had a freezing rate of −32 to −45°C/min, and 10 cm had one of −20 to −29°C/min. Lowercase letters represent significance between columns.

Total post-thaw sperm motility fluctuated widely between 4% and 60% and averaged 23 ± 2% across all CPA and freezing treatments. A significantly higher (β = 0.23, SE = 0.08, *df* = 26, *t* = 2.8, *P* = 0.005) total post-thaw sperm motility was observed using 10% DMFA than 5% DMFA but did not differ significantly from 10% DMSO (24 ± 3% vs 20 ± 2% vs 22 ± 3%, respectively) (Figure 2). FPM ranged from 3% to 6% for frozen–thawed sperm and averaged 5 ± 1% across all treatments, indicating that only 20% of sperm showing movements post-thaw were progressing forward. We did not find any significant difference in FPM by CPA treatment (*df* = 27, 0.03 ≤ *t* ≥ 1.3; *P* > 0.05).

**Figure 3:**
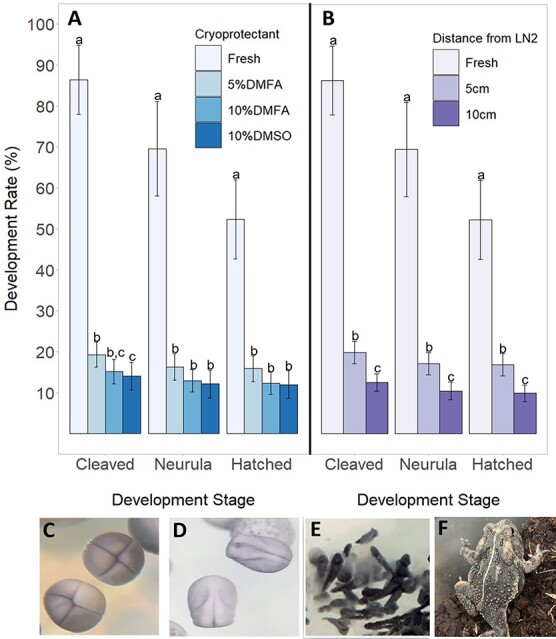
(A) IVF development rate at each stage after fertilization depending on the cryoprotectant added to fresh sperm upon freezing. The data are shown as mean ± SEM, and lowercase letters represent significance between cryoprotectants per development stage. (B) Development rate of individuals at each development stage after fertilization depending on freezing rate, shown as distance from LN_2_. A total of 5 cm from LN_2_ had a freezing rate of −32 to −45°C/min, and 10 cm had one of −20 to −29°C/min. The data are shown as mean ± SEM, and lowercase letters represent significance between freezing rates per development stage. Images of development stages show (C) cleavage, (D) neurulation, (E) hatching and (F) metamorphosis.

We compared fertilization and embryo development results between fresh and frozen–thawed sperm to determine the impacts the cryopreservation process had on sperm functionality. Development of the parthenogenic control (0%) and the amount of cleavage (86 ± 8%), neurula (70 ± 11%) and hatching (52 ± 10%) from the fresh sperm control served as the baseline comparison for post-thaw motility treatments. Parthenogenic controls did not result in any embryo development, so we did not have to account for potential auto-activation in our treatments. Overall, the development of embryos from fresh and frozen–thawed sperm were significantly different for cleavage (*β* = 4.87, SE = 0.35, *df* = 13, *t* = 13.9, *P* < 0.001), neurulation (*β* = 3.73, SE = 0.26, *df* = 13, *t* = 14.3, *P* < 0.001) and hatching (*β* = 2.77, SE = 0.29, *df* = 13, *t* = 9.6, *P* < 0.001), with fresh sperm producing more embryos and tadpoles than frozen–thawed sperm (Fig. 3). Furthermore, total sperm motility had a significant effect on the percent of embryos that cleaved (*β* = 1.59, SE = 0.47, *t* = 3.4, *P* = 0.002). When separated by cryoprotectant, we found that 5% DMFA produced higher cleavage rates than 10% DMSO (*β* = 0.44, SE = 0.18, *df* = 13, *t* = 1.9, *P* = 0.02) but not 10% DMFA (*β* = 0.35, SE = 0.18, *df* = 13, *t* = 2.4, *P* = 0.06) (Fig. 3).

When looking at the effects of freezing rate, we found that sperm frozen at −32 to −45°C/min produced a significantly higher (*β* = 0.32, SE = 0.07, *df* = 26, *t* = 4.8, *P* < 0.001) total post-thaw motility than sperm frozen at −20 to −29°C/min (24 ± 3% vs 19 ± 2%, respectively) (Fig. 2). Furthermore, this faster freezing rate produced a significantly higher (*β* = 0.24, SE = 0.09, *df* = 27, *t* = 2.6; *P =* 0.01) level of post-thaw FPM than the slower freezing rate (5 ± 1% vs 4 ± 1%, respectively). We also found that using sperm frozen at −32 to −45°C/min to fertilize eggs produced significantly higher cleavage (*β* = 0.54, SE = 0.15, *df* = 13, *t* = 3.5, *P =* 0.001), neurulation (*β* = 0.55, SE = 0.16, *df* = 13, *t* = 3.6, *P =* 0.001) and hatching (*β* = 0.58, SE = 0.18, *df* = 13, *t* = 3.3, *P* = 0.002) than sperm frozen at −20 to −29°C/min (Fig. 3). Table 3 shows post-thaw motility of sperm used in IVF and the subsequent fertilization parameters associated with each sperm cryopreservation treatment.

Overall, this study produced 1327 hatched tadpoles from frozen–thawed sperm across all six treatments. These tadpoles were then released back into the wild or kept in captivity for a follow-up experiment to determine growth rate of tadpoles past hatching.

### Experiment 2: the effect of cryopreservation on sperm viability, structural integrity and morphology

**Figure 4:**
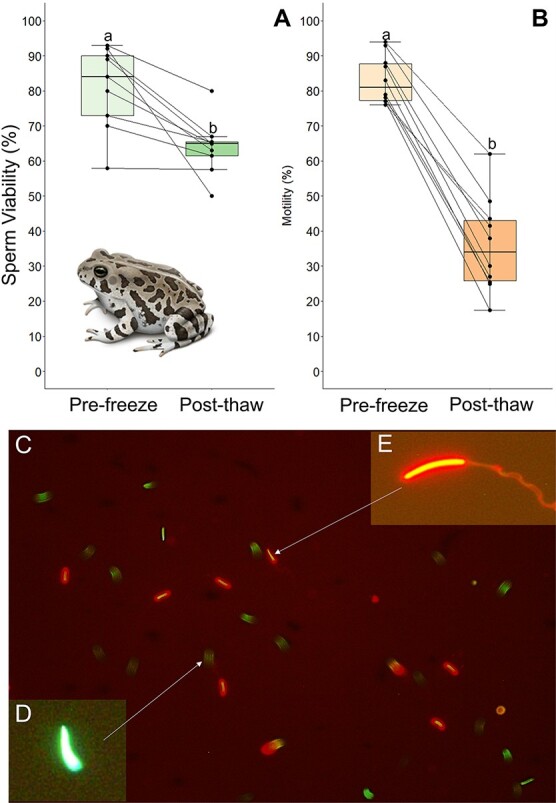
(A) Percentage of sperm viability pre- and post-cryopreservation. Lowercase letters indicate significance between columns. (B) Percentage of sperm motility pre- and post-cryopreservation. Lowercase letters represent significance between columns sperm staining in Fowler’s toad sperm using SYBR-14 and propidium iodide. (C) View from microscope used to measure viability. Sperm that are stained green are live. Sperm that are stained red are dead. (D) Close-up of a live sperm cell. (E) Close-up of a dead sperm cell.

**Figure 5:**
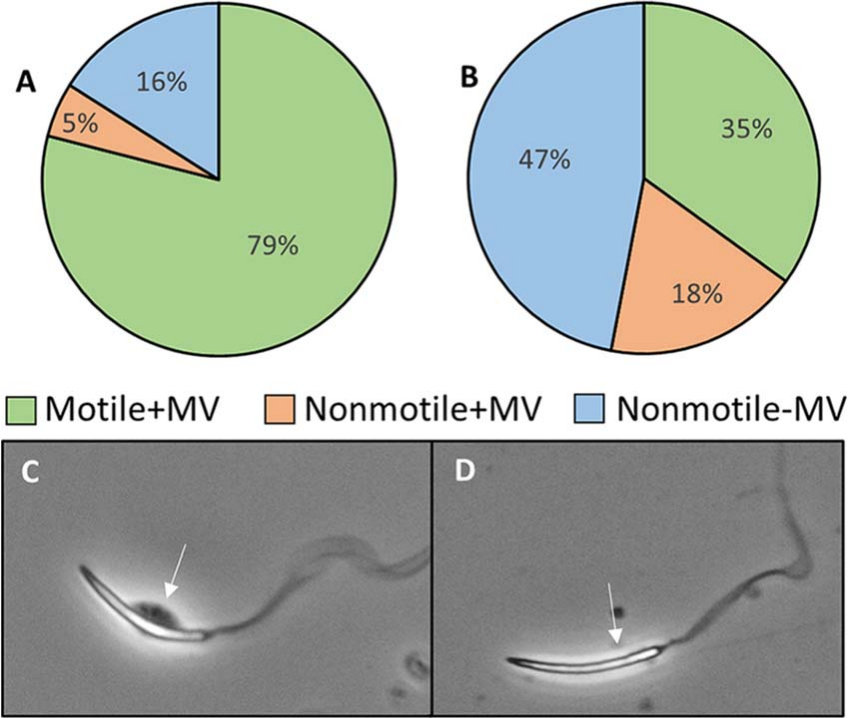
Percentage of motile sperm with the mitochondria vesicle, non-motile sperm with the mitochondria vesicle, and non-motile sperm without the mitochondria vesicle (A) pre-freeze and (B) post-thaw. (C) Sperm cell with the mitochondria vesicle present, as indicated by the arrow and (D) sperm cell without the mitochondria vesicle, as indicated by the arrow.

**Figure 6:**
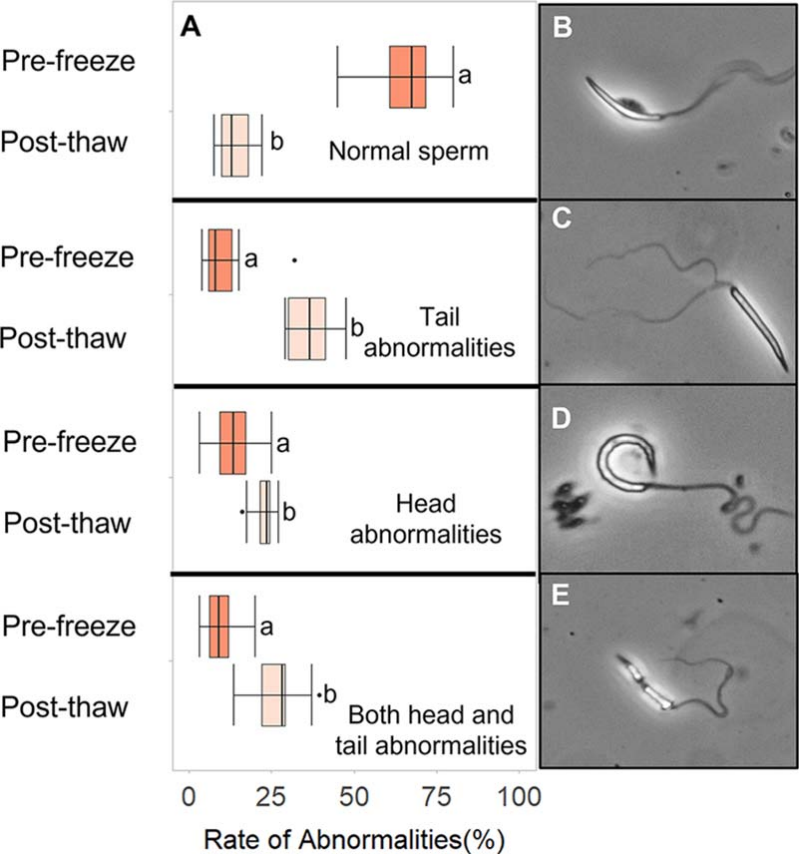
(A) Percentage of sperm with abnormalities pre- and post-cryopreservation. Lowercase letters indicate significance between columns. Images show the different types of abnormalities shown in the graph, including (B) a normal sperm cell, (C) a sperm with both head and tail abnormalities, (D) tail abnormality and (E) head abnormality.

Experiment 2 evaluated how the best cryoprotectant and freezing rate determined from Experiment 1 affect the viability, structural integrity of the MV and morphology of cryopreserved sperm. Table 2 shows the average percent of viable sperm, normal sperm, sperm with abnormal heads, sperm with abnormal tails, sperm with abnormal heads and tails and sperm with MVs present in spermic urine before freezing. Fresh sperm collected for experiment 2 had an average total motility of 83 ± 2%, and frozen–thawed samples had an average motility of 36 ± 4%. Fresh sperm samples contained 52–93% viable spermatozoa, which was significantly higher (*df* = 9, *t* = 2.9, *P* = 0.02) than frozen–thawed samples (78 ± 5% vs 64 ± 2%, respectively) (Fig. 4). We also found that there was a greater (*df* = 14, *t* = 5.8, *P* < 0.001) percentage of viable sperm than motile sperm in post-thawed samples, indicating a higher level of survival post-thaw than suggested by motility (Fig. 4). We found a significantly higher fraction (*df* = 9, *d* = 3.8, *P* = 0.002) of fresh sperm with attached MV than frozen–thawed sperm (80 ± 3% vs 35 ± 4%, respectively), and frozen–thawed sperm had a significantly higher fraction of nonmotile sperm with the MV present (*df* = 9, *d* = 2.5, *P* = 0.006) or absent (*df* = 9, *d =* 3.1, *P* = 0.002) than fresh sperm (19 ± 1% and 47 ± 4% vs 5 ± 2% and 16 ± 3%, respectively) (Fig. 5). Fresh sperm samples contained 45–80% (mean = 66 ± 3%) sperm with normal morphology and low levels of sperm with any type of abnormality (3–32%) (Table 2, Fig. 6A). Overall, fresh sperm samples had a significantly higher (*df* = 9, *t* = 15.5, *P* < 0.001) fraction of normal sperm than frozen–thawed samples (66 ± 3% vs 14 ± 2%), and frozen–thawed sperm samples had a significantly higher number of sperm with abnormal heads (df = 9, *t* = 3.7, *P* = 0.005), tails (*df* = 9, *d* = 3.4, *P* = 0.002) or both (*df* = 9, *t* = 7.2, *P* < 0.001) compared to fresh samples of sperm (23 ± 1%, 37 ± 2% and 27 ± 2% vs 14 ± 2%, 11 ± 3% and 10 ± 2%, respectively) (Fig. 6A).

**Table 2:** Fresh sperm parameters of pooled samples from males selected for freezing and morphology of a normal toad sperm cell

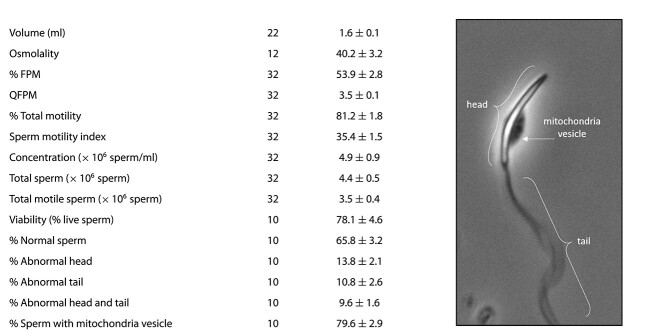

^*^Varying sample sizes is due to the number of individuals used in Experiment 1 vs. Experiment 2

^**^Data are shown as mean ± SEM.

## Discussion

In this study, we developed a cryopreservation protocol that led to the successful recovery of sperm motility, fertilization success, and embryo development using Fowler’s toads as the common animal model for imperilled anuran species. The results indicate that there is a relationship between cryopreservation protocols that lead to higher levels of sperm motility, when compared to the other treatments, and those that lead to higher levels of fertilization success and embryo development, as seen in the faster freezing rate producing the highest percentages of all three parameters and in the significant effect post-thaw total motility had on fertilization success. Because viability data were not collected during the fertilization trials, no determination can be made on how viability affects fertilization. However, as sperm has to be viable (live) in order to be motile, we hypothesize that we will see the same relationship between viability and fertilization success that we do between motility and fertilization success. We also determined that the cryopreservation process led to higher fractions of post-thaw sperm abnormalities, higher numbers of nonmotile spermatozoa without mitochondria vesicles and a lower percentage of viability than pre-freeze sperm, supporting the assumption that cryopreservation negatively affects sperm integrity. Even though cryodamage was noted upon thawing, the frozen–thawed sperm still produced 1327 tadpoles, which is one of the highest number of tadpoles produced by frozen–thawed sperm. This result shows that though cryo-induced abnormalities may be prevalent in sperm, frozen–thawed sperm can still lead to fertilization success and embryo development.

This study is the first to analyse how freezing rate affects fertilization success and embryo development using cryopreserved sperm. Moreover, we show that the faster freezing rate, which produced higher fertilization and development numbers than the slower rate, is the same protocol that produced the highest post-thaw sperm motility. This relationship between these values indicates that post-thaw sperm motility may be a valid representation of the fertilization and embryo development potential of eggs fertilized with frozen–thawed sperm. Knowing optimal freezing rates for improved embryo development is invaluable for managing threatened amphibians that are not breeding well in captive collections. Because the goal of reproductive technologies is to produce offspring that can then develop into reproductively viable adults, it is paramount that we broaden our understanding of how the manipulation of the sperm pre- and post-cryopreservation affects the fertilization and development process. Further research is needed to track development past the tadpole stage and into adulthood to determine any lasting effects the CPAs and freezing rates may have on individuals produced from cryopreserved gametes. Preliminary results from our lab on tadpole growth did not indicate any variation between CPAs and freezing rate up through metamorphosis (Burger, unpublished data).

Most cryopreservation studies to date have frozen sperm from anurans at a slower rate of −5 to −10°C/min (Browne *et al.,* 1998; Clulow and Clulow, 2016; Mansour *et al.,* 2010), yet few have directly compared the effects of varying freeze rates on motility, fertilization, and embryo development. Preliminary experiments conducted by Browne *et al.* (1998) found that a freezing rate of ~ −40°C/min led to < 2% recovered motility and a rate of ~ −30°C/min led to < 10% recovered motility. Similarly, sperm samples from *Atelopus sp*. frozen using a slower freezing rate (samples held 10–13 cm above LN_2_) had a post-thaw motility of ~50–60%, whereas a faster rate (samples held 5 cm above LN_2_) had ~ 20% sperm motility (Naranjo *et al.,* 2021). In contrast, the current study found that a faster rate of ~ −40°C/min (5 cm above LN_2_) led to significantly better post-thaw motility than samples frozen at the slower rate, with some samples reaching nearly 60%. This variation between studies could be caused by the composition of the cryosuspension used to limit cellular damage during the freezing process or species-specific differences in response to the CPAs or freezing rates. Further studies that compare the two freezing rates tested here against the slower rate (−5 to −10°C/min) could further clarify the best freezing method to apply for cryopreservation in Fowler’s toads, with the potential of transferring this protocol to other species, and whether there is opportunity for improvement in post-thaw sperm parameters.

**Table 3:** Summary table of post-thaw FPM and total motility (TM) of sperm used to fertilize eggs in IVF attempts separated by cryoprotectant and freezing rate (5 cm = −32 to −45°C/min; 10 cm = −20 to −29°C/min)

**Treatments**	**Motility of post-thaw sperm for IVF** ^*^			**Fertilization** ^*^				
**CPA**	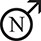	**FPM (%)**	**TM (%)**	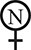	**No. of eggs**	**Cleavage (%)**	**Neurula (%)**	**Hatched (%)**
5% DMFA	6	8.6 ± 1.7^a^	26.3 ± 3.7^a^	6	2612	19.3 ± 4.4^a^	16.3 ± 4.5^a^	15.9 ± 4.5^a^
10% DMFA	6	8.8 ± 2.2^a^	29.9 ± 5.2^a^	6	2865	15.2 ± 4.2^ab^	13.0 ± 3.9^a^	12.3 ± 3.8^a^
10% DMSO	6	7.5 ± 2.1^a^	26.9 ± 6.1^a^	6	2909	14.1 ± 4.7^b^	12.2 ± 4.8^a^	12.0 ± 4.8^a^
**Height**	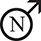	**FPM (%)**	**TM (%)**	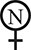	**No. of eggs**	**Cleavage (%)**	**Neurula (%)**	**Hatched (%)**
5 cm	6	9.6 ± 2.3^a^	30.5 ± 5.8^a^	6	4059	19.8 ± 4.7 ^a^	17.1 ± 4.7 ^a^	16.9 ± 4.7^a^
10 cm	6	6.8 ± 1.8^b^	24.1 ± 3.9^b^	6	4197	12.6 ± 3.6 ^b^	10.5 ± 3.7 ^b^	9.9 ± 3.6^b^
**Controls**	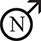	**Motility of sperm**		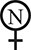	**No. of eggs**	**Cleavage (%)**	**Neurula (%)**	**Hatched (%)**
		**FPM (%)**	**TM (%)**					
Parthenogenic	NA	NA	NA	6	317	0	0	0
Fresh	4	72 ± 8	88 ± 3	4	415	86 ± 8	70 ± 11	52 ± 10

^*^Lowercase letters represent significance between columns within a heading (CPA/height). Values are reported as mean ± SEM.

Another interesting outcome of the IVF attempt is the contrast between the decrease in development seen in embryos fertilized with fresh sperm versus frozen sperm (Fig. 3). In fresh sperm, there seems to be a higher number of embryos that did not develop from one stage to the next, whereas in frozen sperm, there is not much of a decline. The IVF methodology was the same for eggs fertilized with either sperm type, including container size, the number of eggs in each dish and the frequency of water changes. However, because there was a higher number of embryos that developed with fresh sperm, it is possible that this consistency in methodology may have led to the decline in embryo development. According to Seymour and Bradford (1995), respiration of an amphibian embryo in an egg mass is affected by the number of other embryos present in that mass, with more embryos leading to higher rates of oxygen uptake. Because of this, it is possible that the higher number of embryos in fresh sperm treatments were competing for a limited supply of oxygen, while embryos fertilized with frozen sperm did not experience this competition because there were fewer embryos in each dish. Future work could look at measuring the level of oxygen in dishes with multiple versus fewer embryos and potentially supplement dishes with higher oxygen uptake with more frequent water changes or air stones to increase available oxygen for developing embryos.

Our results suggest that either 10% DMFA or 10% DMSO could be used for freezing sperm from Fowler’s toads; both CPAs provided an average of 24% and 22% absolute post-thaw motility, respectively. In particular, we found 10% DMFA as the CPA of choice due to it producing higher levels of post-thaw sperm motility in 14/22 males, with four of these males reaching almost 60%. Furthermore, while 5% DMFA did produce higher levels of cleavage than 10% DMSO, there was no difference between 5% DMFA and 10% DMFA for fertilization or embryo development. This result, combined with the higher post-thaw motility levels, leads to us selecting 10% DMFA as the best CPA. The results described here are similar to preliminary findings from our lab for Fowler’s toad sperm cryopreservation and the need to develop a model species for the conservation of at-risk species (Langhorne, 2016). The cryopreservation protocols developed by Langhorne (2016), and further refined here, have subsequently been applied to anuran sperm freezing by other investigators, including testing in Fowler’s toads (Poo and Hinkson, 2019). Poo and Hinkson (2019) analysed the effect of 5%, 10% and 15% DMFA on post-thaw sperm motility in Fowler’s toads using a constant freezing rate similar to our slower rate (−20 to −29°C/min). In order to accurately compare the two studies, the following discussion focuses on sperm frozen at −20 to −29°C/min in the current study. The optimum cryoprotectant determined by Poo and Hinkson (2019) was 5% DMFA, resulting in ~ 20% average relative (recovered) post-thaw sperm motility, and the current study found a similar recovered motility (22%) when using 5% DMFA. However, unlike Poo and Hinkson (2019), this study found 10% DMFA to be the optimum cryoprotectant, resulting in a recovered motility of 25%. Interestingly, we observed a recovered motility four to five times higher than reported by Poo and Hinkson (2019) when using 10% DMFA at the same freezing rate (25% vs <5%). A possible reason for this variation may be the difference in non-permeating CPAs used in the final cryosuspensions. Both experiments used Trehalose with DMFA or DMSO, yet this study also included BSA in the cryosuspension as a way to further offset the damage done by the freezing process and the toxicity of the permeating CPAs. BSA is a serum albumin protein that has been reported to protect the integrity of post-thaw sperm membranes by eliminating free radicals produced by oxidative stress (Sariözkan *et al.,* 2009; Uysal and Bucak, 2007), increase the fluidity of the sperm membrane to minimize cryodamage (Yamashiro *et al.,* 2006), increase post-thaw sperm motility (Matsuoka *et al.,* 2006; Uysal and Bucak, 2007; Yamashiro *et al.,* 2006), increase fertilization (Sariözkan *et al.,* 2009), maintain acrosomal integrity (Sariözkan *et al.,* 2009; Uysal and Bucak, 2007; Yamashiro *et al.,* 2006) and increase protein concentration of the cryosolution, decreasing ice formation by dehydrating the cell (Tamiya *et al.,* 1985). Thus, the inclusion of BSA in our cryosuspension may have offered additional protection to the spermatozoa, reducing the potential risk of damage normally seen when using higher concentrations of CPAs. More research utilizing BSA as a non-permeating cryoprotectant in anurans could further elucidate the beneficial effects of adding this compound to the freezing matrix.

Following the cryopreservation and thawing process, many surviving spermatozoa exhibit high levels of cellular damage, potentially limiting fertilization success. In anurans, it has been found that the association between the MV and sperm head is particularly weak and prone to rupture or disassociate from the head during the cryopreservation process, rendering the spermatozoa nonmotile (Kouba *et al.,* 2003). As such, relying solely on motility measurements may not provide an accurate representation of the number of spermatozoa that survived the cryopreservation process, since sperm can still be viable but nonmotile due to the loss of the MV. Our results showed that frozen–thawed sperm had high numbers of viable, non-motile spermatozoa, with viability being significantly higher than motility (64% vs 36%, respectively). There was also significantly more non-motile sperm without the MV in post-thaw samples than fresh. These two findings together indicate there are more sperm that survived the cryopreservation process than shown by the motility measurement alone and that sperm may be nonmotile due to the loss of the MV. The separation of the MV from the spermatozoa and the subsequent loss in motility may have severe implications on the fertilization potential of frozen–thawed sperm during IVF attempts. A solution to this problem is intra-cytoplasmic sperm injection (ICSI) for directly injecting the viable, nonmotile sperm into eggs, which could increase IVF success and produce more offspring than current protocols.

In this study, we have shown that toad sperm can be collected from live animals using hormone therapy, cryopreserved, thawed, and used to produce large numbers of offspring through IVF. We report a CPA of 10% DMFA and a freezing rate of −32 to −45°C/min as the optimum parameters for sperm cryopreservation, fertilization success and embryo development. The sperm cryopreservation protocols developed herein have already been applied to species of conservation concern and have been used to produce offspring in the Puerto Rican crested toad (*Peltophyrne lemur,*  Burger *et al.,* 2021), Houston toad (*Anaxyrus houstonensis*, Burger, unpublished data) and Chiricahua leopard frog (*Lithobates chiricahuensis*, Burger, unpublished data). Using this improved protocol to freeze and store sperm in a genome resource bank for reproduction and genetic management could improve sustainability of the afore-mentioned captive breeding programmes, which may lead to increased diversity within animals released to the wild. The widespread application and success of this protocol in producing offspring for other threatened anurans reinforces the use of the Fowler’s toads as a model organism for cryobiology studies that will continue to be refined and improved upon over time. As these studies are further developed, the suite of knowledge on sperm physiology will continue to grow and open up new avenues for research and genetic management with high conservation value to recovery programmes.

## Funding

This work was supported by the Institute of Museum and Library Services (IMLS) National Leadership Grant [grant number MG-30-17-0052-17]. This project was supported by the Mississippi Agricultural and Forestry Experiment Station, the National Institute of Food and Agriculture, US Department of Agriculture, Hatch project under accession number W3173 and the US Department of Agriculture, Agricultural Research Service, Biophotonics project 6066-31000-015-00D.

## Data Availability

The data supporting the study’s findings can be accessed by contacting the corresponding author.
